# DRB1 locus alleles of HLA class II are associated with modulation of the immune response in different serological profiles of HIV-1/Epstein-Barr virus coinfection in the Brazilian Amazon region

**DOI:** 10.3389/fmed.2024.1408290

**Published:** 2024-06-12

**Authors:** Leonn Mendes Soares Pereira, Eliane dos Santos França, Iran Barros Costa, Igor Tenório Lima, Erika Vanessa Oliveira Jorge, Patrícia Jeanne de Souza Mendonça Mattos, Amaury Bentes Cunha Freire, Francisco Lúzio de Paula Ramos, Talita Antonia Furtado Monteiro, Olinda Macedo, Rita Catarina Medeiros Sousa, Felipe Bonfim Freitas, Igor Brasil Costa, Antonio Carlos Rosário Vallinoto

**Affiliations:** ^1^Virology Laboratory, Institute of Biological Sciences, Federal University of Pará, Belém, Brazil; ^2^Postgraduate Program in Biology of Infectious and Parasitic Agents, Institute of Biological Sciences, Federal University of Pará, Belém, Brazil; ^3^Virology Unit, Epstein-Barr Virus Laboratory, Evandro Chagas Institute, Ananindeua, Brazil; ^4^Postgraduate Program in Virology, Evandro Chagas Institute, Ananindeua, Brazil; ^5^Department of Immunogenetics, Hemotherapy and Hematology Foundation of the State of Pará, Belém, Brazil; ^6^Epidemiology and Surveillance Service, Evandro Chagas Institute, Ananindeua, Brazil; ^7^Virology Unit, Retrovirus Laboratory, Evandro Chagas Institute, Ananindeua, Brazil; ^8^School of Medicine, Federal University of Pará, Belém, Brazil

**Keywords:** EBV, HIV-1, coinfection, immune response, HLA class II, DRB1 locus

## Abstract

**Background:**

Epstein–Barr virus (EBV) infection involves distinct clinical and serological profiles. We evaluated the frequency of alleles of locus DRB1 of HLA class II in different serological profiles of EBV infection among HIV-1 infected patients.

**Methods:**

We recruited 19 patients with primary infection, 90 with serological transition and 467 with past infection by EBV, HIV-1 co-infection was 100% in primary infection and approximately 70% in other serological profiles. EBV viral load was quantified by real-time PCR, T lymphocyte quantification and cytokine level analysis were performed by flow cytometry, and HLA locus genotyping was performed by PCR-SSO.

**Results:**

The DRB1*09 allele was associated with primary infection (p: 0.0477), and carriers of the allele showed changes in EBV viral load (p: 0.0485), CD8(+) T lymphocyte counts (p: 0.0206), double-positive T lymphocyte counts (p: 0.0093), IL-4 levels (p: 0.0464) and TNF levels (p: 0.0161). This allele was also frequent in HIV-coinfected individuals (p: 0.0023) and was related to the log10 HIV viral load (p: 0.0176) and CD8(+) T lymphocyte count (p: 0.0285). In primary infection, the log10 HIV viral load was high (p: 0.0060) and directly proportional to the EBV viral load (p: 0.0412). The DRB1*03 allele correlated with serological transition (p: 0.0477), EBV viral load (p: 0.0015), CD4(+) T lymphocyte count (p: 0.0112), CD8(+) T lymphocyte count (p: 0.0260), double-negative T lymphocyte count (p: 0.0540), IL-4 levels (p: 0.0478) and IL-6 levels (p: 0.0175). In the serological transition group, the log10 HIV viral load was high (p: 0.0060), but it was not associated with the EBV viral load (p: 0.1214). Past infection was related to the DRB1*16 allele (p: 0.0477), with carriers displaying IgG levels (p: 0.0020), CD4(+) T lymphocyte counts (p: 0.0116) and suggestive CD8(+) T count alterations (p: 0.0602). The DRB01*16 allele was also common in HIV-1 patients with past EBV infection (p: 0.0192); however, the allele was not associated with clinical markers of HIV-1 infection.

**Conclusion:**

Our results suggest that HLA class II alleles may be associated with the modulation of the serological profiles of the immune response to Epstein-Barr virus infection in patients coinfected with HIV-1.

## Introduction

In the natural history of Epstein–Barr virus (EBV) infection, primary infection is predominantly asymptomatic when it occurs in childhood. However, EBV infection in adolescents and adults can result in infectious mononucleosis, which is characterized by significant clinical variation and can progress to atypical manifestations in approximately 15% of young adults (1, 2). Although the prevalence of primary infection varies between early childhood and early adulthood, it is assumed that on a global scale, approximately 90% of the population will come into contact with the virus before the age of 30 years (3, 4).

Serum levels of the IgM antiviral capsid antigen (VCA) antibody reach their serological peak within the first 5 days of disease onset, a period in which there is an increase in clinical severity and the EBV viral load in the oral mucosa and peripheral blood. Seroconversion to IgG anti-VCA antibodies occurs later, clinical symptoms are still present, and titers tend to remain high and persistent even with a decrease in viral load in the bloodstream (5, 6).

At the beginning of the active phase, the virus infects epithelial cells where it amplifies its viral load, which allows cell-to-cell spread. B cells are subsequently infected due to their expression of CD21, the main receptor for the virus (7). After the end of the active phase of EBV infection, the latent phase begins, in which the viral DNA remains as a closed circular plasmid in the memory B lymphocytes of the host. Coordinated expression of EBV proteins stimulates different patterns of latency that can either induce viral persistence without causing cellular transformation or favor specific lymphoproliferative disorders (8, 9).

The pathology of EBV infection tends to be influenced by coinfection with the HIV-1 virus since the presence of both potentiates the clinical manifestations expected for a monoinfection (10). In fact, the impairment of the immune system caused by HIV contributes to the escape of EBV-infected cells, allowing their proliferation and, eventually, the emergence of EBV-transformed clones (11). These observations are valid even at replication sites in the primary phase of EBV infection, where a relationship between HIV-1 and increased EBV viral load in the tonsils has already been observed (12).

In cases of more severe disease, the emergence of EBV-associated neoplasms correlates with the depletion of specific CD4(+) T lymphocytes induced by HIV-1, and this loss leads to an exhausted CD8(+) T lymphocyte population that can no longer control EBV-mediated lymphoproliferation (13). On the other hand, EBV can also make B lymphocytes susceptible to HIV-1 infection by inducing the expression of CXCR4 and CD4 in these cells (14).

A previous study by our group showed that, of a total of 282 patients from the Brazilian Amazon region with a history of HIV-1 infection evaluated, 19 patients, approximately 7%, were also coinfected with EBV. Coinfection was predominant in homosexual individuals with low education and low family income who used illicit drugs and did not use condoms during their sexual contacts (15).

It has been argued that a hereditary basis for the risk of EBV infection is as relevant as other known risk factors (16). For human leukocyte antigen [HLA], most of the associations observed are related to class I loci.

The genetic profiles of the class I A and B loci have been associated with a polyclonal T lymphocyte response in primary EBV infection, and the specificity of the response can differ slightly, even between similar allelic groups (17). In an *ex vivo* study, maintenance of a balanced anti-EBV response was associated with the HLA-B*08 allele, with restriction to the early BZLF1 protein (18). However, the restricted response of the HLA-A*02:01 allele to epitopes of lytic phase proteins appears to be unstable and associated with the expression of cell death markers (19). Other studies have shown that the microsatellite markers D6S510 and D6S265 and the polymorphisms rs2530388 and rs6457110 in HLA class I loci are associated with changes in viral load and immune cell counts in patients with mononucleosis (20). The strict relationship between EBV and HLA class I is proposed to be due to blockage of synthesis of the TAP transporter by the viral gene BNLF2a, leading to interference with antigen presentation (21).

One study showed that the viral protein EBNA2 downregulates HLA expression through a CIITA transcription factor-dependent pathway (22).

In a detailed search, we found no published studies that investigated the association of HLA locus alleles with HIV/EBV coinfection.

Taken together, the above results show that HLA variability is associated with the elaboration of relevant immunological responses that indicate specific outcomes in EBV infection. In the present study, we evaluated the allelic frequency of HLA class II DRB1 loci and their association with immunological and viral biomarkers in patients with primary EBV infection, in those undergoing serological class transition (interprofiles) and in those with past infection who were predominantly coinfected with HIV-1. Our objective was to identify immunogenetic markers associated with the complex natural history of EBV and/or EBV/HIV-1 coinfection.

## Materials and methods

### Sampling

This was a descriptive, cross-sectional, and analytical study. We recruited patients with symptoms of intermittent fever or episodes of recurrent fever from the Setor de Atendimento Médico Unificado do Instituto Evandro Chagas (Unified Medical Care Sector of Instituto Evandro Chagas; SOAMU-IEC); patients with a positive diagnosis of HIV in a subclinical state from the Centro de Atenção à Saúde nas Doenças Infecciosas Adquiridas (Health Care Center for Acquired Infectious Diseases; CASA DIA); and voluntary blood donors from the Fundação Centro de Hematologia e Hemoterapia do Estado do Pará (Pará State Hematology and Hemotherapy Center Foundation; HEMOPA). From January 2018 to January 2020, peripheral blood samples were collected weekly.

A total of 576 participants were included, of whom 19 had serology consistent with primary EBV infection (P.I.) (anti-VCA IgM (+), anti-VCA IgG (−)), 90 had a class serological transition profile (S.T.) (anti-VCA IgM (+), anti-VCA IgG (+)), and 467 had serology consistent with past EBV infection (Past I.) (anti-VCA IgM (−), anti-VCA IgG (+)).

### Sociodemographic and clinical data

Sociodemographic data (gender, age, education and family income), behavioral data (smoker, alcohol drinker, user of illicit drugs, sexual orientation, active sexual life, steady sexual partner, history of sexual relations with sex workers and use of sexual condoms) and clinical data (sleep quality, diagnosis or family history of cancer, symptoms and history of infections) were obtained through a project-specific questionnaire applied during the participant interview and from medical records databases accessed through authorization filed by the institutions where the study was carried out. All access and disclosure of participant data were included in the study’s ethical opinion.

### Screening and viral load

EBV infection was screened by semiquantitative detection of anti-VCA IgM and IgG class antibodies by enzyme immunoassays (Dia. Pro Diagnostic Bioprobes EBV VCA, Italy). Identification of EBV genotypes was performed by nested PCR targeting the EBNA-3C gene using primers described by Sample et al. (23) and Lorenzetti et al. (24) following appropriate recommendations: (1° round) (F: 5′-AGATGGTGAGCCTGACGTG-3′/R: 5′-GCATCC TTCAAAACCTCAGC-3′) (2° round) (F: 5′-AGAAGGG GAGCGGTGTGTTGT-3′/R: 5′-GGCTGTTTTTGACGTCGGC-3′). The reaction mixture and program were as follows: 10 pmol/μL primers, MgCl2 (50 mM), dNTPs (10 mM), and Taq (5 U/μL); cycling 1° round–1 cycle at 95°C/3′; 20 cycles at 94°C/45, 56°C/45, 72°C/45; 1 cycle at 72°C/7; cycling 2° round–1 cycle at 95°C/3′; 35 cycles at 94°C/45, 56°C/45, and 72°C/45; and 1 cycle at 72°C/7. The presence of a 153-bp fragment was considered positive for EBV-1; a 246-bp fragment was considered positive for EBV-2.

To quantify the EBV load, we used blood plasma samples from patients with positive serology for anti-VCA IgM in a real-time PCR estimation matrix following the protocol of the XGEN MASTER EBV kit (Mobius Life Science, Pinhais, PR, Brazil).

### Cytokine level and cell quantification

Plasma concentrations of the cytokines IL-17A, IFN-γ, TNF, IL-10, IL-6, IL-4 and IL-2 were determined using a Cytometric Bead Array (CBA) with BD FACSCanto™ II and BDTMCBA Human Th1/Th2/Th17 Cytokine kits (BD Biosciences, San Jose, CA, USA). Quantification of CD4(+) T, CD8(+) T, CD4(+)/CD8(+) T (double-positive) and CD4(−)/CD8(−) T (double-negative) lymphocytes was performed by immunophenotyping and flow cytometry using BD FACSCalibur-4 colors and monitoring kits FACSCountTM Reagents and TriTEST™/TruCount (BD Biosciences, San Jose, CA, USA).

### DNA extraction and genotyping of the HLA locus

Peripheral blood samples were collected from the participants, and DNA was extracted using a QiaAmp DNA Mini Kit (Qiagen, Düsseldorf, Nordrhein-Westfalen, Germany) following the manufacturer’s recommendations. The extracted DNA samples were quantified by spectrofluorimetry using Qubit equipment (Invitrogen, USA) following the manufacturer’s recommendations. The degree of purity was evaluated using a NanoDrop™ 2000/2000c spectrophotometer (Waltham, Massachusetts, USA), in which the elution solution used for extraction of genetic material was used as a reference standard.

We standardized the following profiles as the ideal range for successful amplification: concentrations between 10 and 15 μg/ml. The degree of purity for the locus was represented by the ratios 260/280: 1.8–2.0 and 260/230: 1.8–2.2. Samples outside the established standard were diluted in ultrapure distilled water free of DNase/RNase (Invitrogen, USA) at concentrations above the expected concentrations. Samples with concentrations below the expected concentrations were re-extracted.

HLA genotyping was performed using low/medium resolution PCR-SSO methodology (polymerase chain reaction – sequence-specific oligonucleotide) with Luminex technology (Luminex Corporation, Austin, TX, USA) and a LABType^®^ kit (One Lambda Inc., Canoga Park, USA). CA, USA).

The target DNA was amplified by conventional PCR using specific primers for each locus provided by the recombinant Taq kit (Invitrogen, USA) and D-mix solution following the supplier’s amplification protocol, in which, for each sample, 13.8 μL of D-mix, 4 μL of the supplied primer and 0.2 μL of TAQ polymerase, totaling 18 μL of preparation for 2 μL of extracted DNA. Sequence amplification followed the following cycles: 1 cycle at 96°C for 3 min; 5 cycles at 96°C for 20 s, 60°C for 20 s and 72°C for 20 s; 30 cycles at 96°C for 10 s, 60°C for 15 s and 72°C for 20 s; and 1 cycle at 72°C for 10 s. To confirm amplification, electrophoresis was performed on a 2.5% agarose gel.

The amplified DNA was denatured and subjected to hybridization with a set of specific fluorescently stained oligonucleotide probes immobilized on polystyrene microspheres. The reagents were standardized in the following proportions: 2.5 μL of denaturation buffer/sample, 5 μL of neutralization buffer/sample, 34 μL of hybridization buffer/sample, and 4 μL of bead mix/sample.

The microspheres were washed with wash buffer (480 μl/sample) and then reacted with 50 μL of streptavidin-phycoerythrin (SAPE) conjugate, which binds to biotinylated amplified DNA. The SAPE mixture was standardized in the following proportions: 0.5 μL of stock SAPE at 10 × /sample and 49.5 μL of SAPE buffer/sample.

In the analytical phase, the fluorescence intensity of phycoerythrin in each microsphere was evaluated using a Luminex analyzer, and the data were archived. Data analysis was performed with HLA Fusion software to determine the HLA gene alleles, and the reactivity pattern of each DNA sample in relation to the set of probes conjugated to the microspheres allowed the establishment of the genotype.

### Statistical analysis

We applied multivariate analysis to determine the separation of the studied groups according to the values of their variables (lymphocyte quantification, cytokine dosage, anti-EBV antibody titer and EBV viral load). A scatterplot was generated to identify the groups and visualize the group separations and approximations.

We calculated the Spearman coefficient through a matrix of general correlations between the immunological and virological variables in each of the three groups.

The frequency of HLA alleles was calculated by direct counting and compared between groups using the G test. For groups with significantly different allelic profiles, the chi-square residual test was applied to determine the probabilistic importance of each of the alleles, followed by calculation of the odds ratio to determine the advantage or disadvantage of significant alleles. We calculated the false positive probability (FPRP) for significant associations, with a predefined threshold value of 0.5. The odds ratio for calculating the statistical power was 1.5. We adopted a range from 0.25 to 0.00001 as the prior probability of association of the alleles with the serological profiles of EBV infection, in accordance with the recommendations of Wacholder et al. (25).

We also applied the G test to compare sociodemographic and clinical data between the groups. To assess significance, logistic regression was performed, with the EBV serological profile used as the dependent variable and the analyzed data used as independent variables.

HWE was measured by selecting as an alternative hypothesis of interest an excess of heterozygotes in the groups; standard Markov chain parameters were adopted (26).

We applied the Mann–Whitney test for two-by-two comparisons of quantitative and semiquantitative data between the groups and between HLA alleles. We opted for nonparametric tests due to the degree of normality of the variables in question, which was estimated by the Lilliefors test.

We adopted a significance level (α) of 95% while considering a probability of significance (p) less than or equal to 0.05 as a criterion for rejecting the null hypotheses for the statistical analyses. The diagrams and graphs were assembled using GraphPad Prism 8.4.3 (San Diego, CA, USA), and statistical analyses were performed using BioEstat 5.3 software (27).

### Ethical aspects

In compliance with resolutions 466/2012 and 347/05 of the National Health Council, which address guidelines and regulatory standards for research involving humans, the project was submitted for ethical consideration and approved by the Ethics Committee in Research with Human Beings of the IEC (Protocol: 3.121.265; CAAE: 73927717.3.0000.0019). All participants were informed about the research objectives, and those who agreed signed a consent form. Individuals under 18 years of age or who were using any specific therapy (antivirals, antiretrovirals or immunosuppressants) were excluded.

## Results

### Characterization of the groups

Among the sociodemographic and behavioral characteristics, the most common were male sex, age between 18 and 28 years, complete primary education and family income between 1 and 3 years. There were also higher rates of nonsmokers, those who consumed alcohol and those who had no history of using illicit drugs among the participants, as well as heterosexuals and those who had an active sexual life, who had a steady partner, and who had no relationship with sex workers. and who reported using condoms in their relationships (Table 1).

**TABLE 1:** Multifactorial comparison of sociodemographic, behavioral and clinical aspects between patients with primary EBV infection, patients with serological transition and patients with past EBV infection.

Groups	P.I.	S.T.	Past I.	G-test	L.R.	
**Factors**	**n: 19**	**n: 90**	**n: 467**	**p**	**OR (IC 95%)**	***p***
**Sex**						
Female	6 (31.6)	26 (28.88)	108 (23.13)	0.4093	–	–
Male	13 (68.4)	64 (71.11)	359 (76.87)			
**Age**						
18–28	10 (52.6)	40 (44.44)	217 (46.47)	0.4440	–	–
29–39	7 (36.8)	27 (30.00)	129 (27.62)			
40–50	1 (05.3)	18 (20.00)	85 (18.20)			
51–61	1 (05.3)	3 (03.33)	35 (07.49)			
62–72	0	2 (02.22)	1 (00.21)			
**Complete education**						
Illiterate	0	0	1 (00.21)	0.2257	–	–
Literate	2 (10.5)	7 (07.78)	44 (09.42)			
Elementary school 1	6 (31.6)	12 (13.33)	63 (13.49)			
Elementary school 2	9 (47.4)	46 (51.11)	167 (35.76)			
High school	2 (10.5)	20 (22.2)	127 (27.19)			
University education	0	5 (05.56)	65 (13.92)			
**Family income**						
No fixed salary	1 (05.3)	7 (07.78)	19 (04.07)	0.2562	–	–
(<1) salary	8 (42.1)	14 (15.56)	72 (15.42)			
(1–3) salary	10 (52.6)	56 (62.22)	307 (65.74)			
(4–6) salary	0	7 (07.78)	43 (09.21)			
(7–10) salary	0	4 (04.44)	17 (03.64)			
(>10) salary	0	2 (02.22)	9 (01.93)			
**Smoking**						
**history**						
No	10 (52.6)	48 (53.33)	235 (50.32)	0.8500	–	–
Yes	9 (47.4)	42 (46.67)	232 (49.68)			
**Current usage**						
No	6 (66.7)	32 (76.19)	182 (78.45)	0.7162	–	–
Yes	3 (33.3)	10 (23.81)	50 (21.55)			
**Alcohol use**						
**History**						
No	2 (10.5)	12 (13.33)	47 (10.06)	0.6904	–	–
Yes	17 (89.5)	78 (86.67)	420 (89.94)			
**Current usage**						
No	10 (58.8)	39 (50.00)	182 (43.33)	0.2768	–	–
Yes	7 (41.2)	39 (50.00)	238 (56.67)			
**Illicit drug use**						
**History**						
No	14 (73.7)	67 (74.44)	358 (76.66)	0.8814	–	–
Yes	5 (26.3)	23 (25.56)	109 (23.34)			
**Usage time**						
<5 years	3 (60.00)	8 (34.78)	58 (53.21)	0.2663	–	–
≥5 years	2 (40.00)	15 (65.22)	51 (46.79)			
**Sexual orientation**						
Heterosexual	9 (47.4)	48 (53.33)	275 (58.89)	0.7480	–	–
Homosexual	7 (36.8)	30 (33.33)	143 (30.62)			
Bisexual	3 (15.9)	12 (13.33)	49 (10.49)			
**Active sex life**						
No	8 (42.1)	30 (33.33)	137 (29.34)	0.4187	–	–
Yes	11 (57.9)	60 (66.67)	330 (70.66)			
**Fixed partner**						
No	5 (26.3)	41 (45.55)	178 (38.12)	0.2342	–	–
Yes	14 (73.7)	49 (54.44)	289 (61.88)			
**Relationships with sex workers**						
No	16 (84.2)	66 (73.33)	356 (76.23)	0.5770	–	–
Yes	3 (15.8)	24 (26.67)	111 (23.77)			
**Use of condoms**						
No	4 (21.05)	15 (16.67)	105 (22.48)	0.4505	–	–
Yes	15 (78.95)	75 (83.33)	362 (77.52)			
**Difficulty sleeping**						
No	14 (73.68)	49 (54.44)	285 (61.03)	0.2616	–	–
Yes	5 (26.32)	41 (45.56)	182 (38.97)			
**Daily rest hours**						
<8 h	8 (42.11)	59 (65.56)	259 (55.46)	0.0843	–	–
≥8 h	11 (57.89)	31 (34.44)	208 (44.54)			
**Cancer diagnosis**						
No	19 (100.0)	90 (100.0)	465 (99.57)	0.8475	–	–
Yes	0	0	2 (00.43)			
**Family history of cancer**						
No	12 (63.2)	52 (57.78)	261 (55.89)	0.7938	–	–
Yes	7 (36.8)	38 (42.22)	206 (44.11)			
**Degree of kinship**						
First-degree relatives	1 (14.29)	8 (21.05)	41 (19.90)	0.9904	–	–
Second-degree relatives	3 (42.86)	14 (36.84)	73 (35.43)			
Third-degree relatives	3 (42.86)	16 (42.11)	92 (44.66)			
**HIV infection**						
Yes	19 (100.0)	60 (66.67)	317 (67.88)	0.0008	11.4 (1.51–85.88)	0.0064
No	0	30 (33.33)	150 (32.12)			
**History of other infections**						
No history	9 (47.37)	61 (67.78)	330 (70.67)	0.1137	–	–
History	10 (52.63)	29 (32.22)	137 (29.34)			
Syphilis	9 (0.9)	17 (54.84)	80 (54.05)	0.1447	–	–
Gonorrhea	1 (0.1)	7 (22.59)	39 (26.35)			
Other IST	0	7 (22.59)	29 (19.59)			
**Symptomatology**						
Asymptomatic	9 (47.37)	44 (48.35)	253 (54.47)	0.4976	–	–
Symptomatic	10 (52.63)	46 (51.65)	214 (45.53)			
**Symptoms**						
Adenomegaly	0	1 (01.56)	0	0.9312	–	–
Fever	9 (37.50)	19 (29.69)	69 (29.87)			
Sore throat	7 (29.17)	19 (29.69)	77 (33.33)			
Arthralgia	2 (08.33)	5 (07.81)	23 (09.96)			
Headache	4 (16.67)	14 (21.88)	51 (22.08)			
Lymphadenopathy	0	1 (01.56)	0			
Myalgia	2 (08.33)	5 (07.81)	11 (04.76)			

L.R., logistic regression; OR, odds ratio; CI, confidence interval.

In relation to stress and history of cancer, individuals who did not have difficulty sleeping were more likely to experience stress, but with variable rest times and without a diagnosis of cancer or family history (Table 1).

Regarding the history of infections and clinical aspects, individuals with a history of coinfection with HIV and syphilis prevailed. Fever and sore throat were more common in symptomatic individuals (Table 1).

HIV coinfection varied between 70 and 100% in the serological groups, and it was significantly associated with the primary phase of EBV infection (100% of patients; *p* = 0.0008). In fact, patients with primary EBV infection were approximately 11 times more likely to be coinfected with HIV than patients in the other groups were (OR: 11.4; CI (95%): 1.51–85.88; *p* = 0.0064) (Table 1).

**FIGURE 1:**
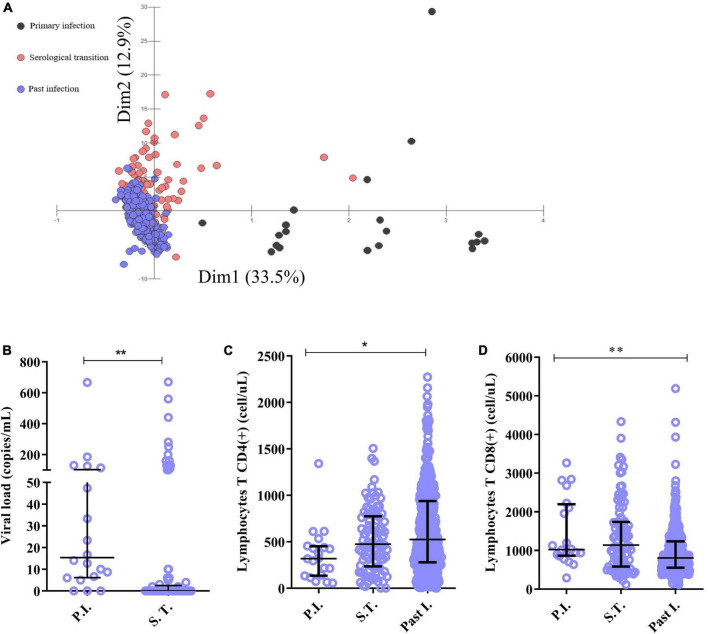
Immunological characterization of the studied groups. **(A)** Discriminant scatter plot of the groups based on immunological and virological aspects. Patients in serological transition and those with past infection tended to overlap; patients with primary infection grouped differently. **(B–D)** Dot plot graphs showing differences in the following predictive immunological and virological variables between the groups: **(B)** viral load, **(C)** CD4+ T lymphocyte count and **(D)** CD8+ T lymphocyte count. *p = 0.005–0.0001; **p < 0.0001.

In multivariate analysis, we observed an overlap between the serological transition groups and the past EBV infection group; the primary infection group tended to cluster in coordinates further away from the others; the generated analysis represented approximately 47% of the total data variance (Figure 1A). The viral load and CD4(+) and CD8(+) T lymphocyte counts were the most relevant parameters. The primary infection group had a greater viral load than did the serological transition group (P.I. = median: 15.33; IIQ: 6.50–78.50); (S.T. = median: 0; IIQ: 0.0–2.0); *p* < 0.00001) (Figure 1B) and a lower CD4(+) T lymphocyte count (P.I. = median: 319.50; IIQ: 140.00–443.25); (S.T. = median: 475; IIQ: 245–764); (Past I. = median: 525.50; IIQ: 280.75–938.25); p: 0.0068) (Figure 1C) but a higher CD8(+) T lymphocyte count than did the other groups (P.I. = median: 1024.50; IIQ: 886.25–2136.75); (S.T. = median: 1140; IIQ: 582–1735); (Past I. = median:807; IIQ: 553–1235); *p* < 0.00001) (Figure 1D).

**FIGURE 2:**
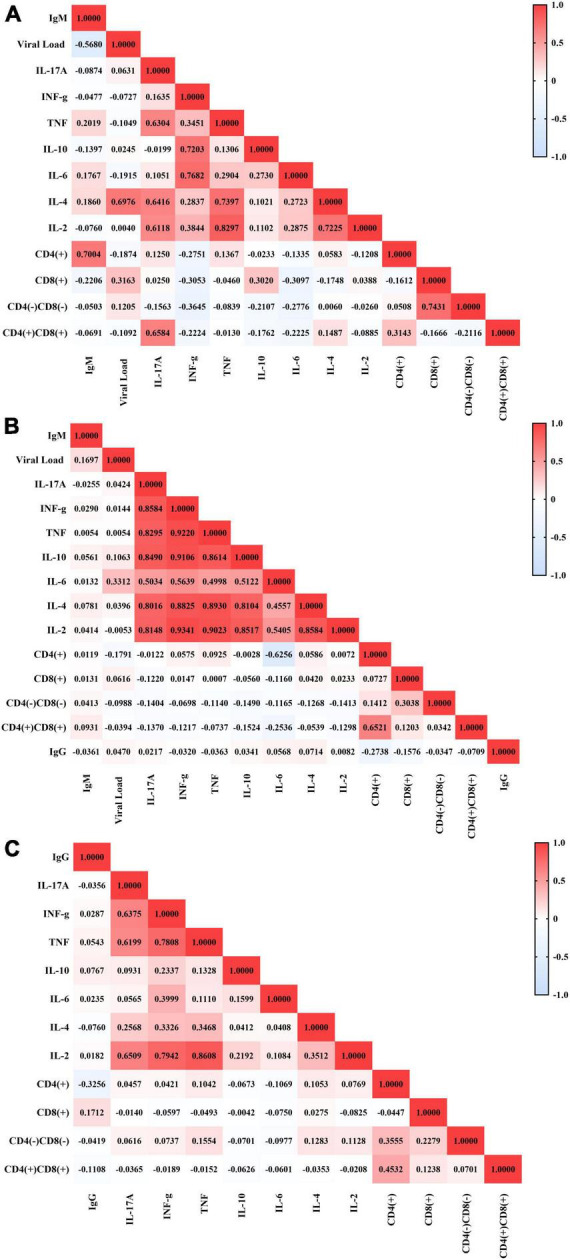
Correlations between immunological and virological factors in the studied groups. Heatmap graphs showing correlation matrices between immunological and virological factors in patients with active infection **(A)**, patients in serological transition **(B)** and patients with past EBV infection **(C)**.

In the group with primary infection (Figure 2A), the IgM concentration correlated negatively with the viral load (r: −0.568; p: 0.0334) and positively with the CD4(+) T lymphocyte count (r: 0.7004; p: 0.0006), IL-6 level (r: 0.1767; p: 0.0457), TNF level (r: 0.2019; p: 0.0522) and IL-4 level (r: 0.1860; p: 0.0517). The EBV viral load correlated negatively with the CD4(+) T lymphocyte count (r: −0.1874; p: 0.0526) and IL-6 level (r: −0.1915; p: 0.0525) but positively with the IL-4 level (r: 0.6976; p: 0.0427). In addition, the CD4(+) T lymphocyte count correlated positively with the IL-17A (*r* = 0.1250; *p* = 0.0494) and TNF (*r* = 0.1367; *p* = 0.0513) levels. The CD8(+) T lymphocyte count correlated negatively with the CD4(+) T lymphocyte count (r: −0.1612; p: 0.0497) and IL-4 level (r: −0.1748; p: 0.0475) and positively with the double-negative T lymphocyte count (r: 0.7431; p: 0.0002). The double-positive T lymphocyte count also correlated positively with the IL-17A level (r: 0.6584; p: 0.0016), and cytokine levels correlated positively with each other (r: 0.1051–0.8297).

In the serological transition group (Figure 2B), the viral load correlated negatively with the CD4(+) T lymphocyte count (*r* = −0.1791; *p* = 0.0569) and positively with the IL-6 level (*r* = 0.3312; *p* = 0.0017). The CD4(+) T lymphocyte count correlated negatively with the IL-6 level (r: −0.6256; p: 0.0021), though the double-positive T lymphocyte count correlated positively with the CD4(+) T lymphocyte count (r: 0.6521; *p* < 0.0001) and negatively with the IL-6 level (r: −0.2536; p: 0.0177). Additionally, cytokine levels correlated positively (*r* = 0.4998–0.9343).

In the group with past EBV infection (Figure 2C), the IgG level correlated negatively with the CD4(+) T lymphocyte (r: −0.3256; *p* < 0.0001) and double-positive T lymphocyte (r: −0.1108; p: 0.0401) counts and positively with the CD8(+) T lymphocyte count (r: 0.1712; p: 0.0016). However, the CD4(+) T lymphocyte count correlated negatively with the IL-6 level (r: −0.1069; p: 0.0476) and positively with TNF (r: 0.1042; p: 0.0535) and IL-4 (r: 0.1053; p: 0.0510) levels and with double-negative T lymphocyte (r: 0.3555; *p* < 0.0001) and double-positive T lymphocyte (r: 0.4532; *p* < 0.0001) counts. The CD8(+) T lymphocyte count correlated positively with the double-positive T lymphocyte (r: 0.1238; p: 0.0219) and double-negative T lymphocyte (r: 0.2279; *p* < 0.0001) counts, and the double-negative T lymphocyte count correlated positively with the IL-2 (r: 0.1128; p: 0.0368), TNF (r: 0.1554; p: 0.0041) and IL-4 (r: 0.1283; p: 0.0175) levels. For the other groups, cytokine levels correlated positively with each other (*r* = 0.0913–0.8608).

### Frequency of the DRB1 locus

**FIGURE 3:**
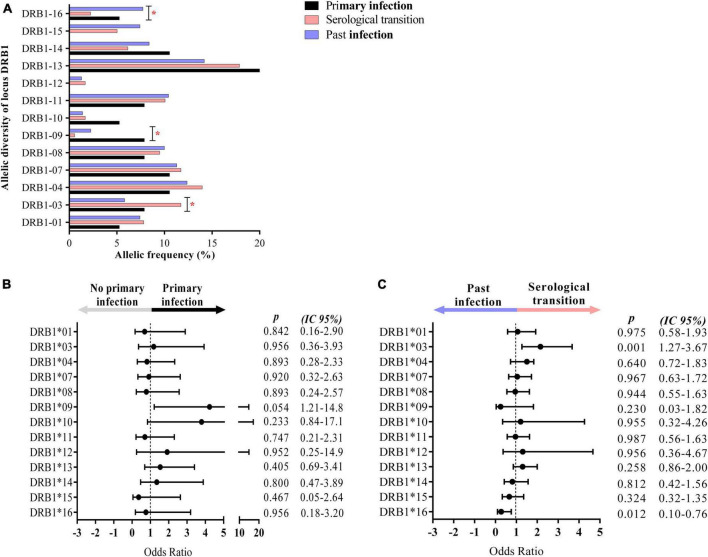
Alleles associated with EBV infection profiles. **(A)** Column graph showing the frequency of alleles at the HLA class II DRB1 locus. The DRB1*09, *03, and *16 alleles were associated with primary infection, serological transition and past EBV infection, respectively. *p = 0.005–0.0001. **(B)** Dot plot graph showing the odds ratio and confidence interval (CI 95%) of DRB1 alleles in primary EBV infection, with the DRB1*09 allele being approximately 4-fold more frequent in the group. **(C)** Dot plot graph showing the odds ratio and confidence interval (CI 95%) of DRB1 alleles at serologic transition and past EBV infection. The DRB1*03 allele was approximately 2-fold more frequent in the serological transition group. The DRB1*16 allele was less frequent in the serological transition group (OR: 0.2724) and approximately 7-fold more frequent in the group with past infection.

The DRB1 locus was in Hardy–Weinberg equilibrium in all groups studied (P.I.: *p* = 0.9158; S.T.: *p* = 0.7626; Past I.: *p* = 0.8574). We observed relevant differences in the frequency of DRB1 locus alleles between the groups studied (*p* = 0.0477), with the DRB1*09 allele showing greater probabilistic relevance in the group with primary EBV infection (residues x^2^: 2.4538), the DRB1*03 allele being more relevant in the serological transition group (residues x^2^: 2.8528) and the DRB1*16 allele being more relevant in the group with past EBV infection (residues x^2^: 2.6223) (Figure 3A and Table 2). The rate of heterozygosity was high in the three groups, with no significant differences among them (*p* = 0.1817) (Table 2).

**TABLE 2:** Allelic frequency of the DRB1 locus of HLA class II in groups with primary infection, in serological transition and with past infection with EBV.

Alleles	Primary		Transition		Past				Primary	Transition	Past
	*N*	%	*N*	%	*N*	%	*p*	G test	residues x^2^ (α : 0.05): 1.96	residues x^2^ (α : 0.05): 1.96	residues x^2^ (α : 0.05): 1.96
*DRB1-01*	2	5.26	14	7.82	69	7.42	0.0477	34.3380	−0.5140	0.2283	0.0233
*DRB1-03*	3	7.89	21	11.73	54	5.81			0.2725	2.8528	−2.7680
*DRB1-04*	4	10.53	25	13.97	115	12.37			−0.3837	0.6206	−0.3997
*DRB1-07*	4	10.53	21	11.73	105	11.29			−0.1597	0.1828	−0.0964
*DRB1-08*	3	7.89	17	9.50	93	10.00			−0.4117	−0.1733	0.3487
*DRB1-09*	3	7.89	1	0.56	21	2.26			2.4538	−1.6167	0.3768
*DRB1-10*	2	5.26	3	1.68	13	1.40			1.8632	0.1250	−0.9673
*DRB1-11*	3	7.89	18	10.06	97	10.43			−0.4938	−0.1111	0.3286
*DRB1-12*	0	0.00	3	1.68	12	1.29			−0.7217	0.4720	−0.1076
*DRB1-13*	8	21.05	32	17.88	132	14.19			1.0636	1.1754	−1.5751
*DRB1-14*	4	10.53	11	6.15	78	8.39			0.5554	−1.0473	0.7166
*DRB1-15*	0	0.00	9	5.03	69	7.42			−1.6934	−1.0253	1.7239
*DRB1-16*	2	5.26	4	2.23	72	7.74			−0.3828	−2.6412	2.6223
Homozygous	1	5.26	16	17.78	54	11.56	0.1817	3.4109	−0.9568	1.8375	−1.2797
Heterozygous	18	94.74	74	82.22	413	88.44			0.9568	−1.8375	1.2797

### The DRB1*09 allele was associated with primary EBV infection

The DRB1*09 allele was approximately 4-fold more frequent in the group with primary EBV infection than in the other groups (OR: 4.235; CI (95%): 1.21–14.80; p: 0.054) (Figure 3B).

**FIGURE 4:**
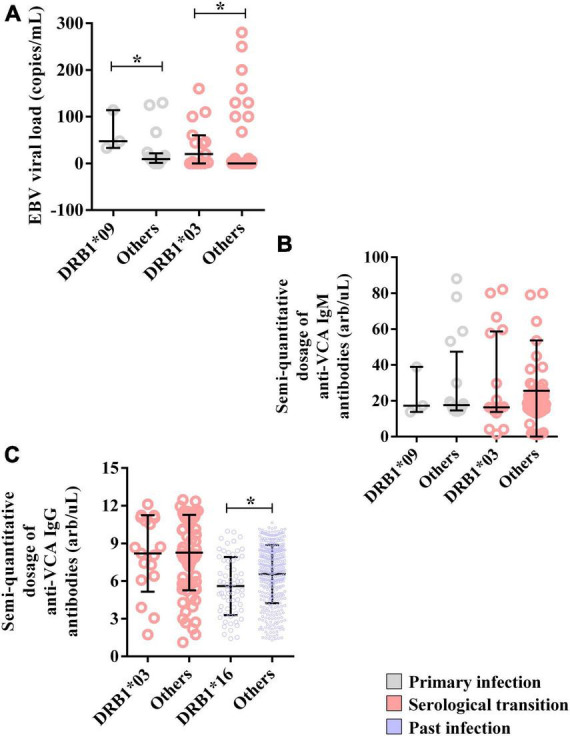
Alleles associated with EBV viral load and anti-VCA IgM and IgG antibody levels. **(A)** Dot plot graph showing the associations of the DRB1*03 and DRB1*09 alleles with the EBV load. **(B)** Dot plot graph showing the lack of association of the DRB1*03 and DRB1*09 alleles with semiquantitative IgM antibody titers. **(C)** Dot plot showing the association of the DRB1*16 allele with the semiquantitative IgG antibody titer. *p = 0.005–0.0001.

The median EBV viral load was greater in carriers of this allele (DRB1*09 = 47.33; IIQ = 40.33–80.67) (others = median: 9.33; IIQ = 3.75–18.33); *p* = 0.0485) (Figure 4A). However, the anti-VCA IgM titer was not associated with DRB1*09 (Figure 4B).

**FIGURE 5:**
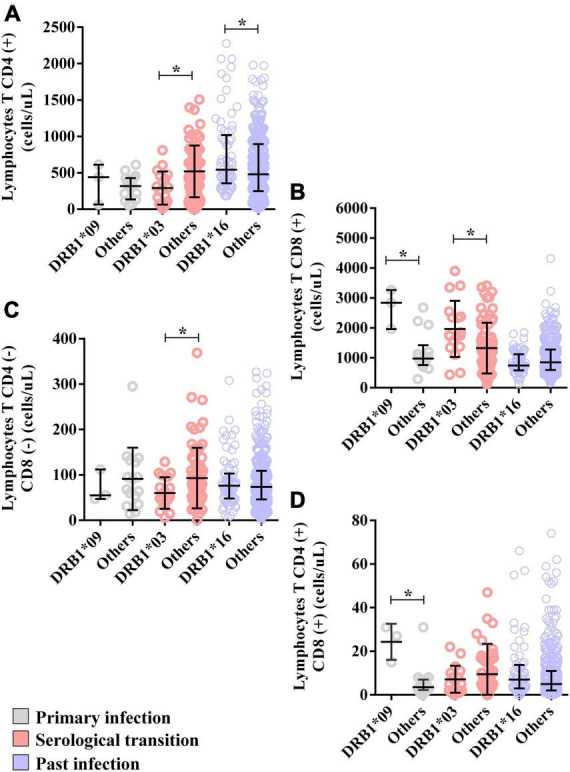
Alleles associated with the T lymphocyte count. **(A)** Dot plot showing the associations of the DRB1*03 and DRB1*16 alleles with the CD4(+) T lymphocyte count. **(B)** Dot plot graph showing the associations of the DRB1*03 and DRB1*09 alleles with the CD8(+) T lymphocyte count. **(C)** Dot plot showing the association of the DRB1*03 allele with the double-negative T lymphocyte count (CD8-CD4-). **(D)** Dot plot graph showing the association of the DRB1*09 allele with the double-positive T lymphocyte count (CD8+CD4+). *p = 0.005–0.0001.

The CD8(+) T lymphocyte count was greater (DRB1*09 = median: 2844; IIQ: 2401.5–3054.0) (others = median: 975.5; IIQ: 795.5–1179.5); *p* = 0.0206) (Figure 5B), as was the double-positive T lymphocyte count (DRB1*09 = median: 27; IIQ: 21–29) (others = median: 3.5; IIQ: 2.75–7.00); *p* = 0.0093) (Figure 5D).

**FIGURE 6:**
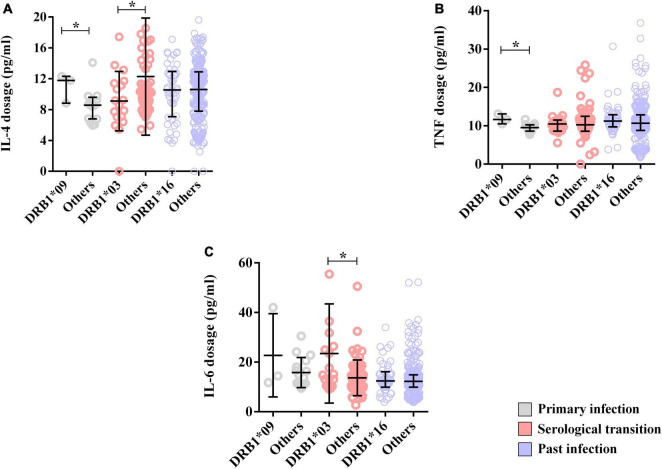
Alleles associated with cytokine dosage. **(A)** Dot plot showing the associations of the DRB1*03 and DRB1*09 alleles with the IL-4 concentration. **(B)** Dot plot showing the association of the DRB1*09 allele with the TNF-α level. **(C)** Dot plot showing the association of the DRB1*03 allele with the IL-6 level. *p = 0.005–0.0001.

The levels of TNF (DRB1*09 = median: 11.64; IIQ: 11.07–12.38); (others = median: 9.52; IIQ: 8.72–10.24); *p* = 0.0161) and IL-4 (DRB1*09 = median: 11.78; IIQ: 10.30–12.04); (others = median: 8.52; IIQ: 6.67–8.85); *p* = 0.0464) were also higher in the DRB1*09 allele carriers (Figure 6C). Other cytokines were not associated with the allele (Supplementary Data 1).

### The DRB1*03 allele was associated with the serological transition profile

The DRB1*03 allele was approximately 2-fold more frequent in patients who underwent a serological transition (OR: 2.1560; CI (95%): 1.27–3.67; p: 0.001) (Figure 3C).

Moreover, the EBV viral load was greater in DRB1*03 allele carriers (DRB1*03 = median: 20; IIQ: 0.00–52.98) (others = median: 0; IIQ: 0–0); p: 0.0015) (Figure 4A). In contrast, anti-VCA IgM and IgG titers were not associated with this allele (Figures 4B, C).

The CD4(+) T lymphocyte counts (DRB1*03 = median: 272; IIQ: 83.25–450.25); (others = median: 477; IIQ: 223.0–743.5); p: 0.0112) (Figure 5A) and double-negative T lymphocyte counts (DRB1*03 = median: 50; IIQ: 39.25–90.00); (others = median: 75; IIQ: 45.5–116.5); p: 0.0540) (Figure 5C) were lower in carriers of the DRB1*03 allele, but the CD8(+) T lymphocyte count was greater (DRB1*03 = median: 1749; IIQ: 1426.00–2367.25); (others = median: 1111; IIQ: 620.0–1687.5); p: 0.0260) (Figure 5B).

Additionally, IL-4 dosage levels were lower in carriers of this allele (DRB1*03 = median: 8.89; IIQ: 7.09–11.34); (others = median: 10.10; IIQ: 8.45–14.10); p: 0.0478) (Figure 6A), but the IL-6 level was greater (DRB1*03 = median: 14.96; IIQ: 11.22–26.00); (others = median: 12.46; IIQ: 9.44–16.62); p: 0.0175) (Figure 6B). Other cytokines were not associated with the allele (Supplementary Data 1).

### The DRB1*16 allele was associated with past EBV infection

In this study, the DRB1*16 allele was significantly associated with past EBV infection (OR: 0.2724; CI (95%): 0.10–0.76; p: 0.012) (Figure 3C), being up to 7-fold more frequent in this group.

The anti-VCA IgG titer was lower in DRB1*16 allele carriers (DRB1*16 = median: 5.41; IIQ: 3.76–7.63) (others = median: 6.9; IIQ: 4.93–8.39); *p* = 0.0020) (Figure 4C).

The CD4(+) T lymphocyte count was greater in carriers of this allele (DRB1*16 = median: 541; IIQ: 361–980) (others = median: 480; IIQ: 250.75–891.25); *p* = 0.0116) (Figure 5A). The counts of other cells were not associated with the allele; however, CD8(+) T lymphocytes tended toward lower counts in carriers of the allele (DRB1*16 = median: 744; IIQ: 595–1106; (others = median: 853; IIQ: 595–1273); p: 0.0602) (Figure 5B).

### HIV infection was associated with the DRB1*09 allele in patients with primary EBV infection

Our data showed that HIV infection was associated with serological profiles of EBV infection, which was more frequent in the group with primary infection (Table 1). We subsequently evaluated whether this aspect was also associated with alleles of the DRB1 locus. We found that the frequency of the DRB1*09 allele was significantly greater in individuals with HIV who were in the primary phase of EBV infection, at approximately 6 times greater in this group (OR: 6.15; CI (95%): 1.79–21.16; p: 0.0067). In contrast, the frequency of the DRB1*16 allele was greater in individuals with HIV who were in the past phase of EBV infection, at approximately 4 times greater in this group (OR: 4.19; CI (95%): 1.27–13.86; p: 0.0192) (Table 3).

**TABLE 3:** Association of the frequency of HLA-DRB1 alleles with HIV infection in different phases of EBV infection.

Alleles	HIV/P.I.		HIV/S.T.		HIV/Past I.				HIV/P.I.	HIV/S.T.	HIV/Past I.	L.R.	
	*N*	%	*N*	%	*N*	%	*p*	G test	Residues x^2^ (α : 0.05): 1.96	Residues x^2^ (α : 0.05): 1.96	Residues x^2^ (α : 0.05): 1.96	*p*	OR (IC 95%)
DRB01*03	3	15.79	13	21.67	41	12.93	0.0023	21.997	0.1776	1.7422	−1.6582	–	
DRB01*09	4	21.05	0	0	13	4.10			3.6938	−1.7810	−0.3776	0.0067	6.15 (1.79–21.16)
DRB01*16	1	05.26	2	03.33	45	14.20			−0.9387	−2.2642	2.5336	0.0192	4.19 (1.27–13.86)
Others alleles	11	57.89	45	75.00	218	68.77			−1.0931	1.0579	−0.3645	–	

L.R., logistic regression; OR, odds ratio; CI, confidence interval.

**FIGURE 7:**
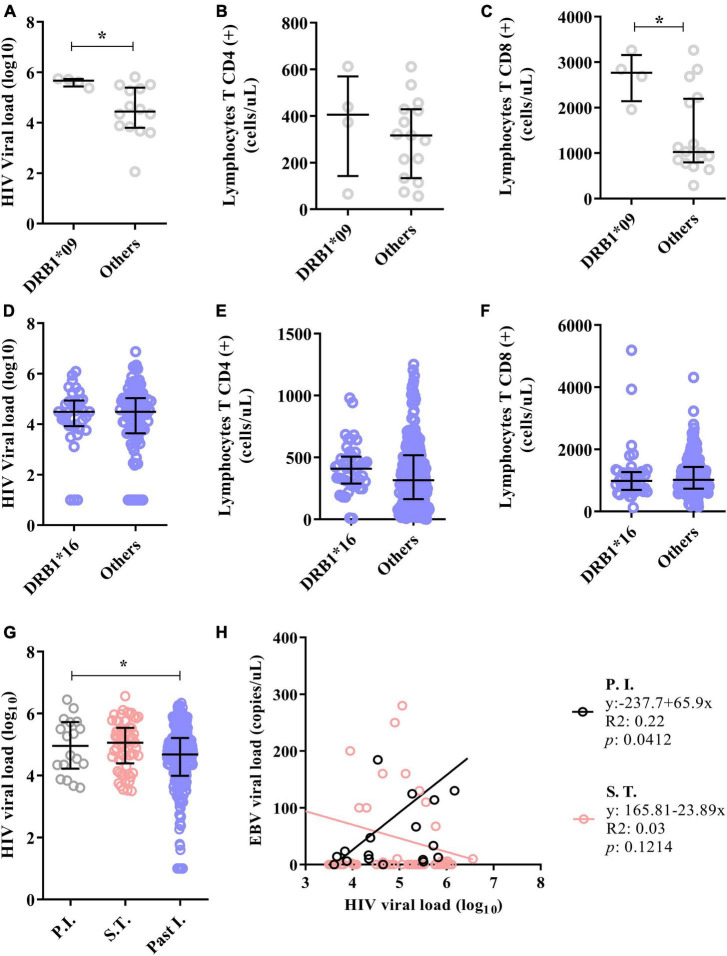
The DRB1*09 allele was associated with HIV/EBV coinfection. **(A)** Dot plot showing the association of the DRB1*09 allele with HIV viral load. **(B)** Dot plot graph showing the lack of association of the DRB1*09 allele with the CD4(+) T lymphocyte count. **(C)** Dot plot showing the association of the DRB1*09 allele with the CD8(+) T lymphocyte count. *p = 0.005–0.0001. **(D–F)** Dot plot graphs showing that the DRB1*16 allele was not associated with markers of HIV infection in individuals with past EBV infection. **(G)** Dot plot showing that the HIV viral load was elevated in the groups with primary EBV infection and in the serologic transition group. *p = 0.005–0.0001. **(H)** Linear regression between the viral loads of both viruses. The HIV viral load correlated positively with the EBV load in patients with primary EBV infection.

We also evaluated whether these alleles are associated with several pathological markers of HIV infection (viral load, CD4+ T-cell count and CD8+ T lymphocyte count) (Figures 7A–H). We observed that carriers of the DRB1*09 allele in the primary phase of EBV infection had both higher log10 HIV viral loads (DRB1*09 = median: 5.67; IIQ: 5.56–5.76); (others = median: 4.45; IIQ: 3.85–5.33); p: 0.0176) (Figure 7A) and CD8(+) T lymphocyte counts (DRB1*09 = median: 2766.5; IIQ: 2506.5–2949.0); (others = median: 1024.5; IIQ: 834.5–2136.8); p: 0.0285) (Figure 7C) than carriers of other alleles. For individuals with past EBV infection and carriers of the DRB1*16 allele, no significant differences were observed in the quantification of these markers (Figures 7D–F).

Our next step was to assess whether the HIV load is associated with the serological profile of EBV infection and the viral load. We found that the log10 HIV viral load was lower in patients with past EBV infection (P.I. = median: 4.96; IIQ: 4.34–5.67); (S.T. = median: 5.06; IIQ: 4.49–5.52); (Past I. = median: 4.68; IIQ: 3.99–5.21); *p* = 0.0060) (Figure 7G).

Furthermore, positive linear regression was inferred between viral load in patients with primary EBV infection (R2: 0.22; p: 0.0412). There was also a negative trend in the viral load in patients who underwent serological transition, although the difference was not statistically significant (R2: 0.03; *p* = 0.1214) (Figure 7H).

### Calculation of the FPRP

The FPRP values for significant findings at different probability levels are shown in Table 4. In most findings, FPRP was notable in the range between 10 and 25% probability of association between alleles with serological profiles of EBV infection, which seems adequate for studies of genetic associations in general (25).

**TABLE 4:** Calculation of the false-positive report probability (FPRP) for risk associations of HLA-DRB1 alleles with serological profiles of EBV infection.

Alleles	Association	OR (IC 95%)	p association	FPRP	p FPRP	Statistical power	Prior probability					
							0.25	0.1	0.01	0.001	0.0001	0.00001
DRB1*09	Primary EBV infection	4.2 (1.21–14.8)	0.054	0.531	0.025397	0.009	0.438	0.501	0.963	0.996	1.000	1.000
	Coinfection HIV-EBV primary infection	6.15 (1.79–21.16)	0.013	0.689	0.004723	0.012656	0.528	0.671	0.974	0.997	1.000	1.000
DRB1*03	EBV serological transition	2.16 (1.27–3.67)	0.001	0.271	0.004644	0.090652	0.133	0.316	0.835	0.981	0.998	1.000
DRB1*16	Past EBV infection	0.27 (0.10–0.76)	0.012	0.528	0.013147	0.043463	0.476	0.731	0.968	0.997	1.000	1.000
	Coinfection HIV-EBV primary infection	4.19 (1.27–13.86)	0.019	0.610	0.018910	0.046188	0.551	0.787	0.976	0.998	1.000	1.000

The low power of the statistical test is challenging if we consider the sampling obtained, suggesting additional validations using larger samples. However, the epidemiological aspects of control studies can lead to bias in the intended analyses.

## Discussion

We stratified the study population into three serological profiles, which were also immunologically distinguished in terms of EBV viral load and CD4(+) and CD8(+) T lymphocyte counts. In patients with primary infection, viral biosynthesis was more active and statistically significant, as we observed CD8(+) T lymphocyte count maintenance and CD4(+) T lymphocyte depletion. This seems to agree with studies showing differential kinetics of T lymphocytes in primary EBV infection, in which the CD4(+) T lymphocyte response appears early, with a rapid decrease, although the CD8(+) T lymphocyte response tends to be more robust and durable (28, 29). This complex response reflects the immunodominance of the proinflammatory cytokine profile in primary infection (30); nevertheless, the presence of anti-inflammatory cytokines suggests the immunomodulation of EBV (31), which could also explain our findings regarding the correlation matrix between immunological factors and cytokines in different profiles.

In this context, we observed that the HLA class II DRB1*09 allele frequency was associated with primary EBV infection. In fact, the association of this allele with the incidence of infection and, mainly, autoimmune diseases in populations with different ethnic profiles has been discussed, and it has been suggested that the allele favors inflammation related to the establishment of pathological conditions (32–36). Recently, a study carried out in Japanese individuals revealed an association between the DRB1*09 allele and severe cases of COVID-19 (37), which, from an immunological point of view, is characterized by a systemic inflammatory state (38). Our results appear to agree with this hypothesis, as carriers of the DRB1*09 allele had high CD8(+) and double-positive T lymphocyte counts and TNF levels, which may be indicative of an attempt to maintain a proinflammatory antiviral response to active EBV infection (39), as represented by the high viral load in carriers. On the other hand, high IL-4 levels in the same group may be associated with viral persistence (40, 41), which is also in line with our results showing a positive correlation between cytokine levels and the EBV load.

However, the negative correlation between the EBV load and CD4(+) T lymphocyte count conflicts with the findings in the literature. This suggests that the observed lymphopenia may, in part, not be specific to EBV infection (42). Interestingly, all patients with primary infection had a history of coinfection, with HIV being the most frequent, and the HIV viral load was high and directly proportional to that of EBV. We believe that the presence of HIV might be closely related to changes in lymphocyte counts, as expected in a typical infection (43), favoring the expansion of EBV (44). In fact, in a recent study involving patients with primary infection and a history of coinfection, we showed that in HIV/EBV coinfection, retroviral pathology benefits at the expense of host immune response maintenance (45).

Here, we highlight that the DRB1*09 allele frequency is associated with HIV-1/EBV coinfection, specifically primary EBV infection, and that both the HIV viral load and CD8(+) T lymphocyte count are elevated in these patients. Again, probable immunological modulation by this allele is related to an attempt to maintain the response to viral coinfection. Our hypothesis is that the overstimulation of active EBV/HIV coinfection highlights the inflammatory response naturally favored by carriers of the DRB1*09 allele in an attempt to modulate a more effective immunological profile in defense against pathogens. Although we did not assess the predominance of response specificity, a consistent response against HIV can trigger immune activation against EBV (46) and may differ in terms of magnitude and polyfunctionality (47). Regardless, there is a strong relationship between the viral loads of the viruses (48).

Patients in serological transition were characterized by a low EBV viral load and maintenance of CD4(+) and CD8(+) T lymphocyte counts; cytokines correlated strongly with each other but without a clear immunodominance profile. This scenario likely reflects sustainable immunological control of the active phase of the infection and consequent management of the homeostatic balance of an expected response in the latent phase (49). In this case, the establishment of a viral memory pool with qualitatively different responses to lytic and latent virus antigens is also observed (50).

In general, patients in serological transition who were coinfected with HIV maintained a high HIV viral load, even though there was no association with an EBV viral load. Hence, the attempt at immune reboot observed in the transition group, which is expected due to the characteristics of EBV in the latent phase (10, 51), may assist in the response to HIV. This is due, among other factors, to the production of CD40 receptor binding protein (CD40L) in CD4(+) T lymphocytes cocultured with EBV-infected B lymphocytes. CD40L can disfavor both the maintenance of EBV replication (51) and HIV replication (52) through the modulation of a consistent immune response. The fact that the HIV viral load was lower in the group with past infection than in the other groups may indicate that this process of immunological control occurs in the long term. Nonetheless, we cannot ignore the close relationship between these viruses and the stimulation of carcinogenic conditions (53), which requires a detailed assessment of long-term aspects of the pathogenesis of coinfection. Most patients in the present study had no cancer diagnosis or family history of cancer.

The DRB1*03 allele was associated with serological transition; however, contrary to the immunological tendency observed in the group, carriers of this allele had a high viral load and a suggestive proinflammatory profile, as represented by an increase in CD8(+) T lymphocytes and IL-6 and low IL-4. Such immune modulation has been associated with the DRB1*03 allele in the context of autoimmune diseases and therapy-induced platelet disorders in different populations (54–59). In addition, a Brazilian ecological study showed the association of haplotypes containing the DRB1*03 allele with the rate of deaths from COVID-19 (60). We propose that the persistence of viral activation specifically in carriers of the DRB1*03 allele can induce an effective immune counterresponse for controlling infection. A longitudinal study evaluating whether this immunological and virological profile of DRB1*03 allele carriers is maintained in the long term, even after complete seroconversion, would be enlightening, especially in cases of active chronic EBV infection (CAEBV) (61).

Patients with past EBV infection had high counts of CD4(+) T lymphocytes but low counts of CD8(+) T lymphocytes. Nonetheless, a certain degree of immunological activity was observed, mainly by the correlation of CD4(+) and double-negative T lymphocytes with proinflammatory cytokines, which may be a reflection of the immune response to HIV infection that was present in approximately 53% of this group. Our results suggest that in the absence of active EBV infection, patients with HIV appear to have a good prognosis, which is in line with studies that reinforce the association of EBV with HIV progression (62, 63).

The DRB1*16 allele was associated with past infection, anti-VCA IgG titer regulation and a high CD4(+) T lymphocyte count but was suggestive of a low CD8(+) T-cell count. Our findings appear to contradict studies that relate this allele to proinflammatory profiles in autoimmune diseases in different ethnic groups, some of which were acquired after viral infections (64, 65). For example, a study associated the DRB1*16 allele with susceptibility to chronic hepatitis B (66). However, we showed that DRB1*16 allele carriers tended to exhibit control of the immune response, consistent with the profile of patients with past EBV infection.

## Conclusion

We conclude that the DRB1*09, *03 and *16 alleles seem to be associated with immunological modulation in different serological profiles of EBV infection in young adult patients from the Brazilian Amazon region who, in the majority, were coinfected with HIV-1. A limiting factor of this proposal is that we were unable to sample patients with only primary EBV monoinfection within the universe studied, so we could actually distinguish whether the frequency of the alleles was more strongly associated with EBV or EBV/HIV-1 coinfection; however, we highlight that, to our knowledge, this is the first report that links these alleles to viral infections.

## Data availability statement

The datasets presented in this study can be found in online repositories. The names of the repository/repositories and accession number(s) can be found in the article/Supplementary material.

## Ethics statement

The studies involving humans were approved by the Ethics Committee in Research with Human Beings of the IEC (Protocol: 3.121.265; CAAE: 73927717.3.0000.0019). The studies were conducted in accordance with the local legislation and institutional requirements. The participants provided their written informed consent to participate in this study. Written informed consent was obtained from the individual(s) for the publication of any potentially identifiable images or data included in this article.

## Author contributions

LP: Data curation, Formal analysis, Investigation, Methodology, Writing – original draft. ESF: Investigation, Methodology, Writing – review and editing. IC: Investigation, Methodology, Resources, Writing – review and editing. IL: Investigation, Methodology, Writing – review and editing. EJ: Investigation, Methodology, Writing – review and editing. PM: Investigation, Methodology, Writing – review and editing. AF: Investigation, Methodology, Writing – review and editing. FR: Investigation, Methodology, Writing – review and editing. TM: Investigation, Methodology, Writing – review and editing. OM: Investigation, Methodology, Resources, Writing – review and editing. RS: Investigation, Methodology, Supervision, Writing – review and editing. FF: Data curation, Investigation, Methodology, Supervision, Writing – review and editing. IC: Funding acquisition, Project administration, Supervision, Writing – review and editing. AV: Conceptualization, Funding acquisition, Project administration, Supervision, Writing – review and editing.
