# Supplementary material for: DRB1 locus alleles of HLA class II are associated with modulation of the immune response in different serological profiles of HIV-1/Epstein-Barr virus coinfection in the Brazilian Amazon region
Source: Front Med (Lausanne). 2024 Jun 12;11:1408290. doi: 10.3389/fmed.2024.1408290 (PMC11199549; doi:10.3389/fmed.2024.1408290)
Supplement: Supplementary file 1 [file Table_1.DOCX]

Supplementary Material

# Supplementary Data 01: Descriptive statistics of quantification of cytokines not associated with alleles of the HLA-DRB1 locus

| **Cytokines** | **P.I.**  **median (IIQ)** | ***p*** |  | **S.T.**  **median (IIQ)** | ***p*** |  | **Past I.**  **median (IIQ)** | ***p*** |
| --- | --- | --- | --- | --- | --- | --- | --- | --- |
| IL-17A |  |  |  |  |  |  |  |  |
| *DRB1*09* | 26.11 (22.81-120.88) | 0.64 | *DRB1*03* | 29.46 (7.57-42.70) | 0.77 | *DRB1*16* | 31.91 (14.94-54.84) | 0.53 |
| *Others* | 31.11 (0-42.70) |  | *Others* | 21.44 (2.11-43.73) |  | *Others* | 28.41 (9.49-51.17) |  |
| IFN-g |  |  |  |  |  |  |  |  |
| *DRB1*09* | 16.35 (10.66-21.77) | 0.37 | *DRB1*03* | 9.61 (8.92-11.23) | 0.44 | *DRB1*16* | 11.59 (9.35-13.24) | 0.34 |
| *Others* | 10.66 (9.93-12.03) |  | *Others* | 10.49 (8.86-12.07) |  | *Others* | 10.92 (8.55-13.35) |  |
| IL-2 |  |  |  |  |  |  |  |  |
| *DRB1*09* | 9.12 (9.04-9.34) | 0.85 | *DRB1*03* | 9.86 (9.04-10.48) | 0.66 | *DRB1*16* | 9.76 (8.03-11.31) | 0.43 |
| *Others* | 9.62 (8.77-10.01) |  | *Others* | 9.42 (8.7-10.46) |  | *Others* | 9.66 (8.25-11.15) |  |
| IL-10 |  |  |  |  |  |  |  |  |
| *DRB1*09* | 11.95 (10.77-12.89) | 0.78 | *DRB1*03* | 11.25 (9.18-12.96) | 0.69 | *DRB1*16* | 10.79 (9.46-13.58) | 0.70 |
| *Others* | 10.71 (10.27-15.71) |  | *Others* | 10.36 (9.22-12.44) |  | *Others* | 11.16 (9.78-12.82) |  |
